# PARP抑制剂对Lewis肺癌细胞及移植瘤放疗增敏作用及其机制

**DOI:** 10.3779/j.issn.1009-3419.2016.01.02

**Published:** 2016-01-20

**Authors:** 维 王, 碧霞 段, 丽 曾

**Affiliations:** 402160 重庆，重庆医科大学附属永川医院肿瘤科 Department of Oncology, Yongchuan Hospital Afliated of Chongqing Medical University, Chongqing 402160, China

**Keywords:** 聚腺苷二磷酸核糖聚合酶抑制剂, 放疗增敏, DNA损伤修复, 凋亡, Poly (ADP -Ribose) polymerase inhibitors, Radiosensitization, Repair of radiation damage, Apoptosis

## Abstract

**背景与目的:**

受电离辐射的肿瘤细胞DNA损伤主要为单链断裂(single strand break, SSB)与双链断裂(double strand break, DSB)，其中SSBs发生的频率数十倍于DSBs，而SSBs多能通过聚腺苷二磷酸核糖聚合酶[Poly(ADP-Ribose) polymerase, PARP]等因子进行修复。相关新药Olaparib(PARP1/PARP2/PARP3抑制剂)靶向作用于细胞SSBs损伤修复，其联合化疗的临床研究取得令人鼓舞结果。本实验旨在研究Olaparib对Lewis肺癌细胞及移植瘤放疗增敏作用，初步探讨其可能机制。

**方法:**

采用MTT法检测Olaparib对Lewis细胞10%抑制浓度(10% inhibitory concentration, IC_10_)值，克隆形成实验验证Olaparib联合放疗的体外增敏作用；成瘤小鼠分为空白对照、Olaparib、放疗(radiotherapy, RT, 2 Gy×5 d)、Olaparib+RT组，动态测量各组移植瘤体积变化；流式细胞术比较各组细胞体外凋亡率，TUNEL法比较移植瘤细胞凋亡；Western blot检测各组DNA损伤相关蛋白γH2AX，凋亡相关蛋白Bax/Bcl-2、Caspase-3表达。

**结果:**

Olaparib对Lewis细胞IC_10_值为4.4 μmol/L，克隆形成实验测得Olaparib放疗增敏比为1.211；移植瘤初体积(处理前)增长4倍所需天数，Olaparib+RT组显著高于单纯RT组(*P* < 0.001)；流式及TUNEL法检测Lewis细胞体内外凋亡率均Olaparib+RT组高于RT组(*P* < 0.05)；Olaparib+RT组细胞及移植瘤中γH2AX、Bax、Caspase-3显著高于RT组，Bcl-2显著低于RT组(均*P* < 0.05)。

**结论:**

Olaparib对Lewis肺癌细胞及移植瘤起到显著放疗增敏作用，其机制可能与增加受照肿瘤细胞DNA双链断裂形成，上调Bax/Bcl-2促凋亡体系蛋白有关。

## Abstract (en)

**Background and objective:**

Te DNA damage of the irradiated tumor cells is mainly single strand breaks (SSBs) and double strand breaks (DSBs), in which the frequency of occurrence of SSBs is dozens of times than DSBs. However, most of the SSBs could be repaired by the Poly (ADP-Ribose) Polymerase (PARP) and other related factors. Recently listed drug-Olaparib (PARP1/PARP2/PARP3 inhibitor) could target the repair pathways of single strand breaks, and recent clinical trials of PARP inhibitors combined with chemotherapy obtained encouraging results. Te aim of this study is to investigate the effect and potential mechanism of radiosensitization of Poly (ADP-Ribose) polymerase inhibitor-Olaparib on lewis cells and xenografs.

**Methods:**

Te inhibition concentration 10% inhibitory concentration (IC_10_) of Olaparib to Lewis cells was detected by methyl thiazolyltetrazolium (MT) assay. Te radiosensitization effect of Olaparib on Lewis cells was determined by classical colony forming assay. Lewis xenografs models were established, and the mice were randomly divided into four groups: Control group, Olaparib group, Radiotherapy group (RT, 2 Gy×5 d), Olaparib combined with RT group. Xenograf volume was measured during the treatment. Flow cytometry was used to analyze the apoptosis rate of the Lewis cells in each group, and the apoptosis of xenograf tissues was observed by TUNEL stain. Te ralative protein levels of γH2AX (associated with DNA strand breaks repair), Bax/Bcl-2, Caspase-3 (apoptosis-associated protein) were detected by Western blot *in vitro* and *in vivo*.

**Results:**

Te IC_10_ value of Olaparib was 4.4 μmol/L. Te radio-sensitivity enhancement ratio (SER) of Olaparib combined with RT was 1.211 *in vitro*. Compared with RT (2 Gy×5 d) alone, the combination of Olaparib with fractionated radiotherapy signifcantly increased the growth delay of Lewis xenografs (*P* < 0.001). Flow cytometry and TUNEL analysis indicated that the apoptosis rate in the combination group was signifcantly higher than in RT group *in vitro* and *in vivo* (*P* < 0.05). Futhermore, Western blot results confrmed that in the combination group the expression levels of γH2AX, Bax, Caspase-3 were increased, while that of Bcl-2 was decreased as opposed to RT group (*P* < 0.05).

**Conclusion:**

Te combination of Olaprib and fractionated radiotherapy can markedly improve the radiobiological effects on lewis cells and xenografs, which may be induced by promoting the formation of DNA double strand break and upregulating the expression of Bax/Bcl-2 pro-apoptotic proteins.

电离辐射所致的肿瘤细胞DNA损伤主要为链断裂：单链断裂(single strand breaks, SSBs)与双链断裂(double strand breaks, DSBs)；其中SSBs发生的频率是DSBs的10倍-20倍^[1]^。在通常情况下，电离单击所致的SSBs多能通过细胞内聚腺苷二磷酸核糖聚合酶[poly(ADPrbose)polymerase, PARP]、DNA连接酶以及其他损伤修复相关因子的参与进行较完整的修复^[2]^，而DSBs的修复则要困难很多，需通过激活同源重组(homologous recombination, HR)或非同源末端连接(nonhomologous end joining, NHEJ)途径完成^[1, 3]^。DSBs成为电离辐射在细胞染色体上最致命的损伤，DNA损伤修复与否则是影响受照细胞存活的关键因素^[1, 4]^。基于PARP在细胞SSBs修复中的关键性作用，靶向抑制其修复的新思路成为近年来研究的热点之一，相关新药Olaparib(PARP1/PARP2/PARP3抑制剂，奥拉帕尼)于2014年12月美国FDA批准上市。临床研究^[5-7]^结果表明，Olaparib联合紫杉醇、吉西他滨、铂类等化疗药物治疗可使得患者获益。目前，PARP抑制剂在联合放疗方面研究较少，已有的相关研究报告^[8, 9]^显示，其在体外联合照射细胞可提高放疗生物效应。本实验在Lewis肺癌细胞及小鼠移植瘤模型上研究PARP抑制剂联合放疗时生物效应的变化，并探索其可能的机制。

## 1 材料与方法

### 1.1 材料

#### 1.1.1 细胞株与实验小鼠

Lewis肺癌细胞株为本实验室保存。C57/BL小鼠：雌性，6周-8周龄，重15 g-20 g，饲养于无特定病原体(specific pathogen free, SPF)级环境，购于北京华阜康生物科技公司，实验动物许可证编号：SCXK(京)2014-0004。实验获本院动物保护和应用委员会批准，动物实验人员资格证书编号：CQLA-2013-0734。

#### 1.1.2 主要试剂

MTT试剂盒购于北京康为世纪生物科技公司，Annexin V-FITC/PI双染细胞凋亡检测试剂盒购于Biosciences公司，Olaparib-AZD2281购于Selleck Chemicals公司，BCA蛋白浓度测定、Beyo ECL Plus发光试剂购于上海碧云天生物技术公司，A nt i-Phospho -Hi stone H2AX(Ser139)购于Upstate公司，Bax、caspase-3、Bcl-2兔抗鼠单克隆抗体购于Abcam公司，TUNEL试剂盒购自美国罗氏公司。

### 1.2 细胞培养及移植瘤小鼠建模

Lewis肺癌细胞培养于DMEM/HIGHGLUCOSE培养基中(10%胎牛血清，100 μg/mL青霉素-链霉素)，于37 ℃、5%CO_2_、90%湿度孵箱中培养，细胞贴壁。取对数期生长细胞，消化计数，约1.5×10^6^个细胞/只接种于小鼠右侧肋部。

### 1.3 MTT检测Olaparib IC_10_浓度

Lewis细胞以8.0×10^3^个/孔接种于96孔板，细胞贴壁，各孔加入Olaparib[浓度梯度(μmol/L)：0、3.125、6.25、12.5、25、50]，各浓度3个复孔。培养48 h后各孔中加入20 µL MT，避光4 h弃上清，加入150 µL二甲基亚砜，避光振荡5 min。酶标仪测各孔吸光度(density, D)值(570 nm波长处)。存活率=(实验组各孔D值/空白组D值) ×100%，通过BLISS法计算10%抑制浓度(10% inhibitory concentration, IC_10_)与50%抑制浓度(50% inhibitory concentration, IC_50_)值。IC_10_作为后续Olaparib体外用药浓度。

### 1.4 克隆形成实验比较

实验分为放疗(radiotherapy, RT)组与Olaparib+RT组，两组照射均含0 Gy、0.5 Gy、1 Gy、2 Gy、4 Gy、6 Gy剂量点。均使用Synergy直线加速器6 MV X射线(剂量率：1 Gy/min)等中心照射，射野10 cm×15 cm，源皮距=100 cm，瓶面放一1.5 cm厚等效有机玻璃。取对数期细胞，根据不同照射剂量点以100个-10, 000个细胞/孔(表 1)接种于6孔板，待细胞贴壁后，用药组照射前2 h加入Olaparib后行相应剂量点照射。受照细胞培养10 d后，经固定、染色、晾干处理，采用光镜计数各剂量点集落数目(≥50个细胞为一集落)。接种效率=0 Gy点平均集落数/100，存活分数(survival fraction, SF) =该剂量点平均集落数/(该点接种细胞数×接种效率)，根据经典单击多靶模型SF=1-[1-exp(-D/D_0_)]^N^拟合曲线，得到平均致死剂量D_0_值，放疗增敏比(sensitivity enhancement ratio, SER) =D_0_(RT组)/D_0_(Olaparib+RT组)。

**1:** 各剂量点细胞存活分数 Cell survival fraction at each dose point

Dose (Gy)	Numbers (cells)	Average number of colony			SF	
		RT	Olaparib		RT	Olaparib+RT
0	100	74.333	67.666		1.000	1.000
0.5	120	72.333	64.000		0.810	0.788
1	200	73.000	58.333		0.491	0.431
2	600	69.666	49.666		0.156	0.122
4	2, 000	125.000	35.666		0.084	0.026
6	10, 000	66.333	21.000		0.008	0.003
SF=Average number of colony/(number of cells inoculated×efficiency of inoculation). SF: survival fraction.						

### 1.5 流式细胞术检测体外细胞凋亡

实验分为空白对照、Olaparib、RT(2 Gy)、Olaparib+RT(2 Gy)组，细胞以3.5×10^5^个/孔密度接种于6孔板上培养24 h(用药组照射前2 h加入Olaparib)，接受2 Gy照射。培养24 h，消化、洗涤、离心收集细胞，加入200 µL缓冲液重悬，再加入5 µL Annexin V/FITC和10 µL碘化丙啶，避光孵育15 min后加入300 µL缓冲液待用。使用流式细胞仪分类计数。右上象限及右下象限分别代表晚期与早期凋亡细胞。

### 1.6 小鼠移植瘤外照射

小鼠移植瘤体积平均长至250 mm^3^后，随机分为4组(*n*=5)：空白对照、Olaparib、RT(2 Gy×5 d)、Olaparib+RT组，0.9%Nacl或Olaparib (用法用量依据预实验结果及已有研究资料^[10]^：50 mg/kg/d，灌胃，照射前30 min-60 min给药)。空白对照(0.9%Nacl, d1-d5)、Olaparib组(Olaparib, d1-d5)、RT组(移植瘤照射，2 Gy/d，d1-d5)、Olaparib+RT组(Olaparib，d1-d5；移植瘤照射，2 Gy/d，d1-d5)。每隔2天游标卡尺外部测量移植瘤最大径A及最小径B，瘤块体积V=π/6×[(A+B)/2]^3^，绘制肿瘤生长曲线，移植瘤长至4倍初体积(1, 000 mm^3^)后处死小鼠。比较各组小鼠移植瘤长至4倍初体积(1, 000 mm^3^)时所需天数。

### 1.7 Western blot检测γH2AX、Bax/Bcl-2、Caspase-3蛋白表达

收集目的细胞(2 Gy剂量点，受照后2 h)、瘤块组织(小鼠放疗结束后，即d6)，RIPA裂解液裂解细胞(组织)，调整样品蛋白浓度。8%SDS-PAGE分离样品蛋白60 µg，250 mA恒流电转膜至硝酸纤维滤膜，封闭2 h，置于一抗封闭液中，4 ℃孵育过夜。将膜置于含辣根过氧化物酶偶联的IgG二抗中，孵育2 h后采用ECL Plus发光试剂显色。以GAPDH为内参，采用Image Lab软件分析条带灰度值，以目的蛋白/GAPDH比值半定量反应蛋白相对表达水平。

### 1.8 TUNEL法检测瘤块细胞凋亡

采用凋亡细胞的原位末端转移酶标记法(TUNEL法)，TdT酶液和荧光标记液按1:9比例混合，配成TUNEL反应液(整个操作步骤按说明书进行)。正常细胞核染为蓝色，凋亡细胞核染为棕黄色，在×400高倍视野(high power, HP)光学显微镜下随机选取5个癌区，计数凋亡细胞数(个/HP)。

### 1.9 统计学方法

使用IBM SPSS 19.0统计软件进行数据分析。数据以Mean±SD表示，两组数据间比较使用*t*检验，两组以上数据比较使用单因素方差分析(*ANOVA*)。以*P* < 0.05为差异有统计学意义。

## 2 结果

### 2.1 Olaparib对Lewis细胞增殖抑制作用

MTT检测结果示，Lewis细胞在浓度梯度(μmol/L)为：0、3.125、6.25、12.5、25、50的Olaparib作用下存活百分比依次为：100%、97.33%、84.36%、59.67%、39.16%、24.56%，计算得出IC_10_与IC_50_值分别为：4.4 μmol/L、19.6 μmol/L。可见随Olaparib浓度增高，细胞存活率降低，即Olaparib体外对Lewis细胞存在剂量毒性，故选择低毒剂量IC_10_值作为Olaparib体外用药浓度。

### 2.2 细胞克隆形成情况

克隆形成实验得出，RT组与Olaparib+RT组细胞在0 Gy、0.5 Gy、1 Gy、2 Gy、4 Gy、6 Gy各剂量点存活分数(表 1)。多靶单击模型SF=1-[1-exp(-D/D_0_)]^N^拟合曲线(图 1)得出放射生物学指标D_0_值(Gy)在RT组与Olaparib+RT组中分别为：0.797、0.658。Olaparib联合放疗SER为1.211(0.797/0.658)。

**1:**
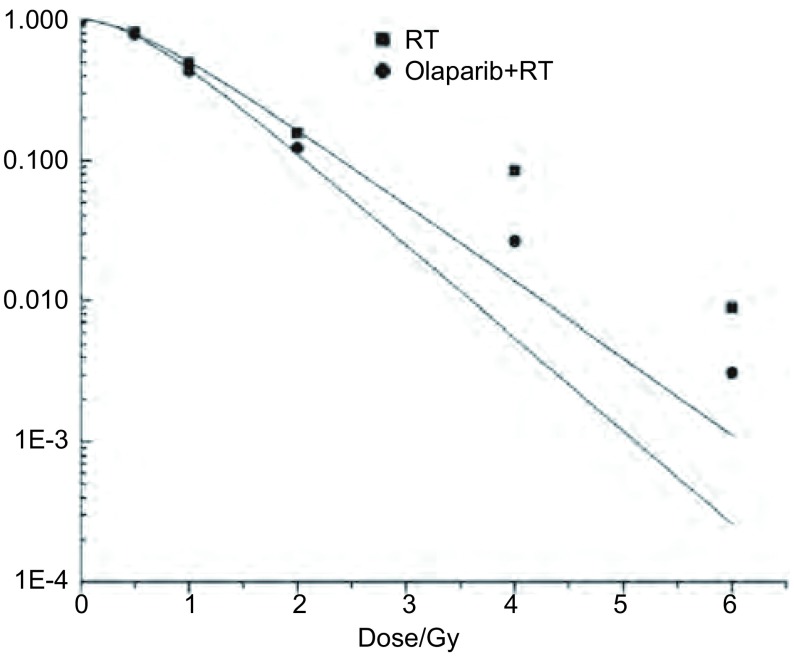
多靶单击模型拟合细胞存活曲线 Cell survival curves plotted by the "single-hit multi-target model". RT: Radiotherapy.

### 2.3 不同处理组细胞体外凋亡差异

细胞接受2 Gy照射后24 h，行流式细胞术检测细胞凋亡率(图 2)。Olaparib+RT组、Olaparib组、RT组、空白组中Lewis细胞凋亡率依次为：(24.77±2.0) %、(3.89±1.1) %、(16.22±2.4) %、(0.53±0.1) %。可见Olaparib+RT组细胞凋亡率显著高于其余三组(均*P* < 0.05)。

**2:**
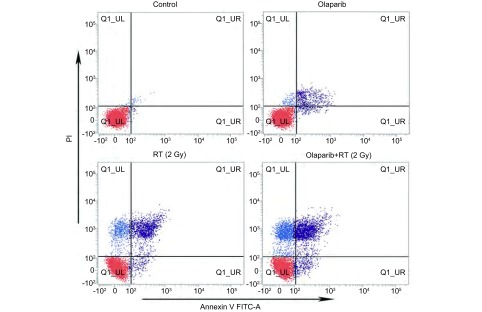
Lewis细胞体外凋亡率差异。流式细胞术检测各组细胞凋亡率，可见Olaparib+RT组细胞体外凋亡率显著高于其余三组（均*P* < 0.05）。 Differential cell apoptotic rate *in vitro*. Cell apoptotic rate was measured by flow cytometry analysis. The apoptotic cells in Olaparib+RT group were significantly higher than the other three groups *in vitro* (all *P* < 0.05).

### 2.4 不同处理组移植瘤细胞体内凋亡差异

光镜下(× 400) Olaparib+RT组、Olaparib组、RT组、空白组移植瘤组织中TUNEL染色阳性(凋亡)细胞的细胞核呈棕黄色(图 3)，其平均凋亡细胞数(个/HP)依次为：(12.4±1.1)、(4.4±1.1)、(8.0±1.2)、(2.2±0.8)。统计得出，Olaparib+RT组细胞凋亡率显著高于其余三组(均*P* < 0.05)。

**3:**
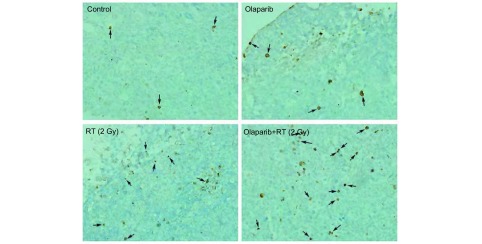
Lewis细胞体内凋亡率差异。TUNEL染色检测各组细胞凋亡情况（×400）。Olaparib+RT组细胞体内凋亡率显著高于其余三组（均*P* < 0.05）。 Differential cell apoptotic rate *in vivo*. Cell apoptotic rate in xenograft tissues was observed by TUNEL stain (×400). The apoptotic cells in Olaparib+RT group were significantly higher than the other three groups *in vivo* (all *P* < 0.05).

### 2.5 DNA损伤及凋亡相关蛋白表达差异

Lewis细胞在接受2 Gy照射后2 h与移植瘤照射(2 Gy×5 d)后1天，Western blot检测各处理组γH2AX(DNA双链断裂相关蛋白)，促凋亡蛋白Bax、Caspase-3，抗凋亡蛋白Bcl-2表达。结果显示，Olaparib+RT组与RT组相比，γH2AX、Bax、Caspase-3蛋白在体外实验中分别上调60.5%、40.0%、42.0%，在移植瘤中分别上调71.4%、37.7%、31.3%；Bcl-2体外实验中下调45.2%，移植瘤中下调61.0%(图 4)。

**4:**
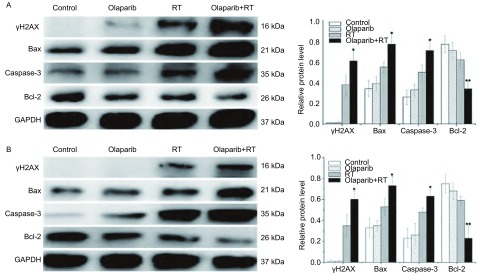
Lewis细胞体内外DNA损伤及凋亡相关蛋白表达。A：Lewis细胞体外照射（2 Gy）后；B：Lewis移植瘤照射结束（2 Gy×5 d）后，Western blot检测γH2AX、Bax、Caspase-3、Bcl-2蛋白表达。^*^P < 0.05，^**^P < 0.01相较于RT组。 DNA damage and apoptosis related protein expression of Lewis cells *in vitro* and *in vivo*. The ralative protein levels of γH2AX, Bax/Bcl-2, Caspase-3 were measured by Western blot. A: After Lewis cells were irradiated *in vitro* (2 Gy); B: After Lewis xenografts were irradiated (2 Gy×5 d). ^*^P < 0.05, ^**^P < 0.01 *vs* RT group.

### 2.6 移植瘤生长曲线比较

各处理组小鼠用药及放疗过程中精神饮食状态正常，无明显全身性不良反应，各组移植瘤体积平缓长至250 mm^3^左右，开始相关实验。各组移植瘤增长过程中均有出现1个-2个移植瘤中央部破溃。10 Gy剂量照射结束后同时剥取未破溃瘤块行前述组织相关指标检测(图 5A)。在Olaparib+RT组、Olaparib组、RT组、空白组中移植瘤增长至4倍初体积(1, 000 mm^3^)所需时间(d)依次为：(29.6±3.8)、(11.6±2.6)、(21.2±2.3)、(10.8±1.1)(图 5B)。可见，Olaparib+RT组移植瘤长至4倍初体积所需天数显著高于其余三组(均*P* < 0.05)。

**5:**
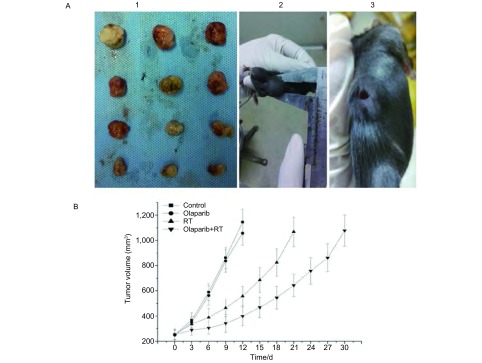
荷瘤小鼠与移植瘤生长延缓曲线。A1：Lewis移植瘤照射（2 Gy×5 d）后剥取瘤块用于相关指标检测；A2：荷瘤小鼠（未破溃）；A3：荷瘤小鼠（瘤块中心破溃）；B：移植瘤增长至4倍初体积（1, 000 mm^3^）生长延缓曲线。 Tumor-bearing mice and Lewis xenografts growth delay curves. A1: Xenografts were isolated for detecting at the next day after irradiated (2 Gy×5 d); A2: Tumor-bearing mice without xenograft rupture; A3: Tumor-bearing mice with xenograft rupture; B: Delay curves of Lewis xenografts increasing to quadruple size from the start.

## 3 讨论

根据经典的"单击多靶"模型，电离辐射可致肿瘤细胞内产生大量的SSBs，同样的，作用于DNA化学结构的细胞毒类药物也可使肿瘤细胞产生较多SSBs。多数的SSBs能通过PARP等相关因子修复，当PARP活性被靶向抑制后，大量外源性的SSBs存在于细胞内，当进入下一个复制叉后，SSBs可转变为DSBs^[11]^。修复DSB，必须通过激活HR或NHEJ信号通路来完成，若该通路中的关键分子(如BRCA1/2、ATM等)发生功能突变，即可对细胞产生所谓的"合成致死"效应，这就是Olaparib及其他PARP抑制剂在临床研究中单药或联合化疗治疗伴有BRCA1/2突变的恶性肿瘤的基础。

凋亡是肿瘤细胞受电离辐射后产生的主要生物效应之一，在线粒体介导的凋亡途径中，细胞在生存信号缺失、DNA严重受损(如DSB)、生长因子缺乏等情况下，可通过Bcl家族的促凋亡成员(如Bax等)与抗凋亡成员(如Bcl-2等)调控线粒体膜通透性，释放凋亡活性物质进入细胞质，引起Caspase级联反应，诱导细胞凋亡^[12]^。与正常组织细胞相比，DNA损伤修复通路在肿瘤细胞中过度激活，并且在机体接受放化疗时，对其有着负性调控作用^[13]^。本实验旨在研究Olaparib联合放疗时，是否可通过增加Lewis细胞DNA损伤及凋亡而起到放疗增敏作用。

本实验首先在体外细胞水平研究Olaparib联合放疗时的增敏效应。运用MTT技术检测Olaparib对Lewis细胞的增殖抑制作用，可见随药物浓度增加，抑制率上升，即Olaparib单药对Lewis细胞存在细胞毒作用，故选择低毒剂量IC_10_值作为体外联合放疗时的药物浓度。通过放射生物学研究常用的克隆形成实验，比较了单纯放疗与Olaparib联合放疗在Lewis细胞层面的生物效应差异，得出Olaparib联合放疗组D_0_值较RT组增加了21.1%，SER为1.211，证实了Olaparib体外的放疗增敏效应。为进一步探究其可能机制，检测不同处理组与受照细胞DSBs相关的敏感指标，即与DSBs量存在对应关系的磷酸化组蛋白H2AX(γH2AX)，其被认为是检测DNA双链断裂的"金标准"。通过蛋白印迹相对定量得出，γH2AX在空白及单药Olaparib组中未表达，而在RT与Olaparib+RT组中高表达，且联合组γH2AX表达量较RT组增加约60%。可见，体外Lewis细胞在低剂量Olaparib联合放疗时即可显著增加DSBs形成。

实验进一步通过流式细胞术检测不同处理组细胞的凋亡率得出，Olaparib联合放疗组较单放疗组细胞凋亡率显著提高。研究^[14-16]^表明，Bax与Bcl-2蛋白可形成异源二聚体抑制凋亡，Bax间可形成同源二聚体促进凋亡，即在Bax/Bcl-2途径中Bax促进凋亡而Bcl-2抑制凋亡，并进一步通过细胞色素C及Caspase-9等因子激活下游Caspase-3蛋白，最终诱导细胞凋亡。本研究对这些凋亡相关蛋白检测发现，Lewis细胞在接受2 Gy照射后，Olaparib联合放疗组促凋亡蛋白Bax及Caspase-3较单纯放疗显著增加，抗凋亡蛋白Bcl-2显著下降。Wesierska等^[17]^研究PARP-1抑制剂AZD2261发现，其可显著抑制乳腺癌MCF-7与Skbr-3细胞克隆形成，并通过Caspase-3蛋白的激活诱导细胞凋亡。在本实验中，Olaparib单药组对Lewis细胞凋亡影响较小(约3.89%)，考虑与用药浓度较低相关(IC_10_值浓度)。Tuli等^[18]^研究PARP1/2抑制剂ABT-888联合放疗胰腺癌MiaPaCa-2细胞时发现，ABT-888单药(10 μmol/L)时对MiaPaCa-2细胞凋亡相关蛋白Caspase表达影响小，而当联合放疗时可显著提高促凋亡蛋白表达。这与本研究结果相一致。

在Lewis细胞移植瘤模型实验中，Olaparib单药给予5 d较空白对照组并未引起明显的肿瘤生长延缓，而当联合放疗(2 Gy×5 d)后，观察到移植瘤显著的生长延缓效应，与单纯放疗组相比，其增长至4倍初体积所需天数多出近10 d。同体外研究类似，实验检测了受照射后移植瘤组织中凋亡细胞、DNA损伤及凋亡相关蛋白的表达情况，获得了与体外研究一致的结果，即联合组较单纯放疗组移植瘤组织中凋亡细胞更多，DSBs相关γH2AX、促凋亡蛋白Bax与Caspase-3表达更高，抗凋亡蛋白Bcl-2表达下调，进一步验证了体内外Olaparib联合放疗时的增敏效应。

综上所述，本研究通过Lewis肺癌细胞及移植瘤模型证实了Olaparib的放疗增敏效应，其可通过增加受照Lewis细胞的DSBs形成，上调Bax/Bcl-2促凋亡体系蛋白表达而提高照射生物效应。为PARP抑制剂在联合放疗方面的应用提供了实验依据，为临床肺癌放疗提供一个新的优良的放疗增敏剂，值得进一步的体内及临床研究。
